# Central Role of IL-23 and IL-17 Producing Eosinophils as Immunomodulatory Effector Cells in Acute Pulmonary Aspergillosis and Allergic Asthma

**DOI:** 10.1371/journal.ppat.1006175

**Published:** 2017-01-17

**Authors:** Evelyn Santos Guerra, Chrono K. Lee, Charles A. Specht, Bhawna Yadav, Haibin Huang, Ali Akalin, Jun R. Huh, Christian Mueller, Stuart M. Levitz

**Affiliations:** 1 Department of Medicine, University of Massachusetts Medical School, Worcester, MA, United States of America; 2 Department of Pathology, University of Massachusetts Medical School, Worcester, MA, United States of America; 3 Horae Gene Therapy Center and Department of Pediatrics, University of Massachusetts Medical School, Worcester, MA, United States of America; Rutgers Biomedical and Health Sciences, UNITED STATES

## Abstract

*Aspergillus fumigatus* causes invasive pulmonary disease in immunocompromised hosts and allergic asthma in atopic individuals. We studied the contribution of lung eosinophils to these fungal diseases. By *in vivo* intracellular cytokine staining and confocal microscopy, we observed that eosinophils act as local sources of IL-23 and IL-17. Remarkably, mice lacking eosinophils had a >95% reduction in the percentage of lung IL-23p19^+^ cells as well as markedly reduced IL-23 heterodimer in lung lavage fluid. Eosinophils killed *A*. *fumigatus* conidia *in vivo*. Eosinopenic mice had higher mortality rates, decreased recruitment of inflammatory monocytes, and decreased expansion of lung macrophages after challenge with conidia. All of these functions underscore a potential protective role for eosinophils in acute aspergillosis. Given the postulated role for IL-17 in asthma pathogenesis, we assessed whether eosinophils could act as sources of IL-23 and IL-17 in models where mice were sensitized to either *A*. *fumigatus* antigens or ovalbumin (OVA). We found IL-23p19^+^ IL-17AF^+^ eosinophils in both allergic models. Moreover, close to 95% of IL-23p19^+^ cells and >90% of IL-17AF^+^ cells were identified as eosinophils. These data establish a new paradigm in acute and allergic aspergillosis whereby eosinophils act not only as effector cells but also as immunomodulatory cells driving the IL-23/IL-17 axis and contributing to inflammatory cell recruitment.

## Author Summary

The opportunistic fungus, *Aspergillus fumigatus*, causes a spectrum of diseases ranging from invasive aspergillosis in the severely immunosuppressed to allergic asthma in atopic individuals. Here we explored the contribution of eosinophils, a type of white blood cell, to host defenses and pathogenesis in murine models of invasive pulmonary aspergillosis and asthma. We found eosinophils co-produce the cytokines IL-23 and IL-17 in both aspergillosis models as well as a model of OVA-induced asthma. Eosinophils killed the conidia (spores) of *A*. *fumigatus* in vivo and mice that lacked eosinophils were more susceptible to invasive aspergillosis. These observations suggest eosinophils play a more prominent role in defenses against invasive pulmonary aspergillosis than heretofore appreciated and identify eosinophil-derived IL-23 and IL-17 as potential therapeutic targets in allergic asthma.

## Introduction

*Aspergillus fumigatus* is an opportunistic mold that produces conidia that are both small (2.5–3 μm in diameter) and readily airborne [1]. These characteristics make *A*. *fumigatus* conidia easily dispersible, while also promoting access to the alveolar spaces in the human airway [2]. It is estimated that on average, individuals inhale hundreds of conidia a day [3]. Despite such frequent exposure, in immunocompetent hosts *A*. *fumigatus* is rarely pathogenic. Its ability to cause disease is dependent on the immunological status of the host. Thus, in immunocompromised individuals, particularly those with quantitative or qualitative phagocyte defects; conidia can germinate and invade lung parenchyma resulting in a highly lethal infection known as invasive aspergillosis (IA). It is estimated that >200,000 people develop life-threatening IA annually [4]. In atopic patients and about 2–15% of patients with cystic fibrosis, sensitization to *A*. *fumigatus* can lead to allergic reactions that can drive asthma pathogenesis and lead to allergic bronchopulmonary aspergillosis (ABPA) [2,5]. Globally, approximately 5 million people suffer from ABPA [6].

Although eosinophilia is a hallmark of several allergic diseases including ABPA and severe asthma with fungal sensitization (SAFS) [7,8], comparatively less is known about the involvement of eosinophils in acute aspergillosis. O’Dea et al. correlated levels of fungal cell wall chitin with eosinophil recruitment to the lungs in response to repeated aspiration of *A*. *fumigatus* conidia [9]. Lilly et al. [10] have shown that ΔdblGATA-1 mice (which lack eosinophils) infected with the ATCC 13073 strain of *A*. *fumigatus* conidia suffer from higher fungal burdens than WT mice. These studies also linked eosinopenia to lower levels of IL-17A two days post-infection [10]. IL-17A exists as a disulfide-linked homodimer and binds with high affinity to the IL-17RA/RC complex. IL-17A also forms a disulfide-linked heterodimer with IL-17F [11,12]. IL-17F can exist as a homodimer as well, binding the same IL-17R complex. For clarity, the IL-17RA/RC ligands will be referred heretofore as IL-17, unless otherwise specified.

IL-17 production is either induced or augmented by IL-23, which is a heterodimeric cytokine composed of IL-23p19 and IL-12p40 subunits. The relationship between IL-23 and IL-17 is known as the IL-23/IL-17 axis [11]. IL-23 is among a group of cytokines that activate signal transducer and activator of transcription (STAT)-3 [13].

*A*. *fumigatus* readily elicits IL-23 and IL-17 production from the lungs after exposure [14]. Although recognized primarily for the induction of neutrophilia, pro-inflammatory cytokines such as IL-6 and IL-1β, and the up-regulation of antimicrobial peptides [15], IL-17 has been reported to induce the recruitment of eosinophils in a model of chronic aspergillosis [16]. High levels of IL-17 have also been correlated with symptom severity in allergic asthma [17]. A remarkably large number of innate and adaptive immune cell types has been reported to be capable of producing IL-17, including γδ T cells, invariant natural killer T cells, type 3 innate lymphoid cells (ILC3s), neutrophils, macrophages, CD8^+^ T cells (T_c_17) and CD4^+^ T cells (T_H_17) [12,15,18,19,20].

The cellular source of IL-23 has primarily been studied in connection to T_H_17 development. In the context of this paradigm, antigen presenting cells such as dendritic cells and macrophages have been identified as its main sources [11]. Here, we show that eosinophils are a local source of IL-23 and IL-17 in response to both acute *A*. *fumigatus* infection and in two different asthma models. In addition, we show that eosinophils are able to associate with and kill *A*. *fumigatus* conidia. We also investigate their function as immunomodulators in acute aspergillosis by regulating the recruitment of inflammatory monocytes and the expansion of macrophages in the lungs. Finally, we describe their ability to confer protection against mortality in acute aspergillosis. Taken together, our findings support a paradigm whereby eosinophils and eosinophilic production of IL-23 and IL-17 are beneficial to defenses against invasive aspergillosis but deleterious to the host in allergic disease.

## Results

### Eosinophils as sources of innate IL-23p19 and IL-17A in acute pulmonary aspergillosis

**Fig 1:**
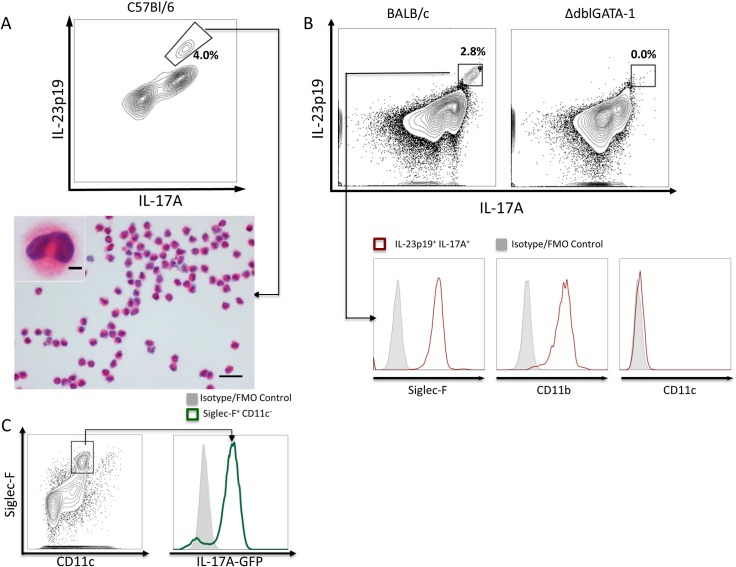
Eosinophils as sources of IL-23p19 and IL-17. (A) C57Bl/6 mice were infected with 5x10^7^ AF293 conidia and then treated 2 hours later with 500 μg intraperitoneal (IP) monensin. Mice were euthanized 8 hours post-infection, lung single-cell suspensions were made and ICS performed on fixed and permeabilized cells with anti-IL-23p19-eFluor 660 and anti-IL-17A-PECy7. FACS analysis uncovered a population producing both cytokines. That population was sorted and stained with H&E, revealing cells consistent with eosinophil morphology (i.e. polymorphous nucleus and pink cytoplasm). Scale bar corresponds to 20μm (and 3μm in figure inset). FACS plot representative of 3 experiments, each of which had 2 mice. (B) An IL-23p19^+^ IL-17A^+^ population was also detected in BALB/c mice infected with 5x10^7^ AF293 conidia, which was almost completely absent in ΔdblGATA-1 mice. Further characterization of the IL-23p19^+^ IL-17A^+^ population by surface markers confirmed they were eosinophils (Siglec-F^+^ CD11b^+^ CD11c^-^). FACS plots representative of 3 experiments with 3 mice per group. (**C**) IL-17A-GFP reporter mice were infected with 5x10^7^ AF293 conidia. Two days post infection, eosinophils (Siglec-F^+^ CD11c^-^) were assessed for the expression of GFP. FACS plot is representative of 3 separate experiments.

*A*. *fumigatus* has been shown to stimulate the IL-23/IL-17 axis before adaptive responses are mounted [14]. Therefore, we sought to identify innate sources of the components of the IL-23/IL-17 axis in the lungs after challenge with *A*. *fumigatus* conidia. By *in vivo* intracellular cytokine staining (ICS) we uncovered a cell population co-producing IL-23p19 and IL-17A in the lungs of C57Bl/6 mice within the first eight hours of infection with 5x10^7^ *A*. *fumigatus* conidia (Fig 1A). To identify the cell type(s) responsible for their production, the IL-23p19^+^ IL-17A^+^ population was sorted 8 h post-infection (Fig 1A). In accordance with eosinophil morphology, ≈95% of the sorted cells stained strongly with eosin in their cytoplasm, and their nuclei were characteristically polymorphous [21]. We also assessed the IL-23p19^+^ IL-17A^+^ population in BALB/c mice acutely infected with *A*. *fumigatus* and found the cells were Siglec-F^+^ CD11b^+^ and CD11c^-^ (Fig 1B). This pattern of surface marker expression has been shown to accurately define eosinophils in the murine lungs [22]. However, in mice where eosinophilopoiesis is disrupted (ΔdblGATA-1; BALB/c background), IL-23p19^+^ IL-17A^+^ cells were largely absent (Fig 1B).

To further confirm eosinophils express IL-17A, we infected IL-17A^GFP/GFP^ mice in the C57Bl/6 background. Due to the autofluorescence exhibited by eosinophils and alveolar macrophages in the channel used to detect GFP signal [23,24], we stained cells with a monoclonal antibody against GFP conjugated to Alexa Fluor 647. We found that eosinophils (Siglec-F^+^ CD11c^-^) indeed expressed GFP in the IL-17A reporter mouse line (Fig 1C). IL-23 and IL-17 production is not a universal feature of all fungal stimulated eosinophil populations as co-incubation of bone marrow-derived eosinophils (BM-eos) with *A*. *fumigatus* conidia, zymosan or LPS elicited levels of these cytokines that were below the lower limits of detection as measured by an ELISA that was sensitive to 4 pg/ml. In addition, there was no signal over baseline when the stimulated BM-eos were examined by ICS. Levels of IL-23 and IL-17 remained undetectable even following stimulation of the bone marrow-derived eosinophils with IL-5, IL-1β, GM-CSF, IL-17E, PGE_2_, transforming growth factor-β and IL-6 over the time and concentration ranges stated in *Materials and Methods*. Moreover, stimulation with IL-23 failed to elicit detectable IL-17 (<4 pg/ml) and stimulation with IL-17AA did not result in detectable IL-23 (<4 pg/ml).

**Fig 2:**
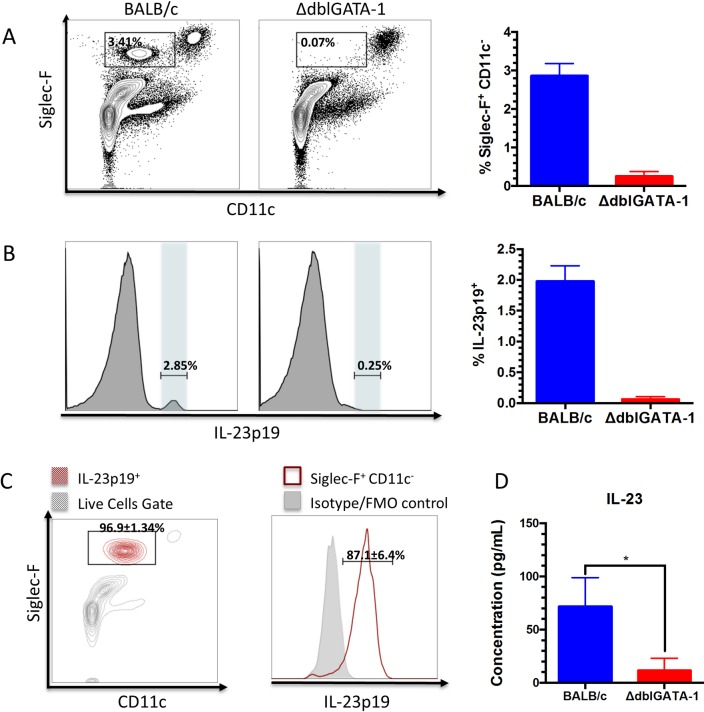
Eosinophils are a significant source of IL-23p19 in acute aspergillosis. (A) ΔdblGATA-1 mice lack eosinophils (Siglec-F^+^ CD11c^-^) after infection with 5x10^7^ AF293 conidia when compared to BALB/c mice. Left panel shows representative FACS plots whereas right panel shows mean ± SEM (n = 5–6 mice/group) percentage of Siglec-F^+^ CD11c^-^ cells. P < 0.0001 comparing groups. (B) Intracellular IL-23p19 staining of lung cells of AF293-infected BALB/c and ΔdblGATA-1 mice after treatment with monensin shows fewer IL-23p19^+^ cells in the absence of eosinophils 8h post infection. Left panel shows representative FACS plots whereas right panel shows mean ± SEM (n = 5–6 mice/group) percentage of IL-23p19^+^ cells P < 0.0001 comparing groups. (C) ICS was performed on BALB/c mice infected with AF293 as in B. Left panel shows IL-23p19^+^ gate (maroon) overlaid onto all live cells (gray) in the Siglec-F vs. CD11c window. The plot shows the mean ± standard deviation (SD) of IL-23p19^+^ events found within the eosinophil gate (Siglec-F^+^ CD11c^-^). Right panel shows the shift in the IL-23p19 channel of the eosinophil population compared to FMO/isotype control in a representative histogram. The mean ± SD of Siglec-F^+^ CD11c^-^ cells which were also IL-23p19^+^ is shown in the plot. Each plot is representative of 2 separate experiments with a total n = 5 mice. (D) BALF from BALB/c and ΔdblGATA-1 mice were collected 60 h after challenge. IL-23 levels, as measured by an ELISA specific for the IL-23p19/IL-12p40 heterodimer, were significantly reduced (p < 0.05, n = 6/group) in eosinopenic mice.

To our knowledge, this is the first report showing that eosinophils express IL-23p19. To further explore this finding, we looked for pulmonary IL-23 sources by comparing eosinophil-deficient ΔdblGATA-1 and wild-type BALB/c mice challenged with *A*. *fumigatus* conidia (Fig 2A). In comparison to BALB/c mice, ΔdblGATA-1 mice exhibited a >95% reduction in the percentage of IL-23p19^+^ cells detected in the lungs (Fig 2B). Nearly all (96.9%) IL-23p19^+^ cells in WT mice were found to be in the eosinophil (Siglec-F^+^ CD11c^-^) gate (Fig 2C). Moreover, the majority (87.1%) of eosinophils recruited to the lungs were IL-23p19^+^ (Fig 2C). As functional IL-23 is comprised of IL-23p19 and IL-12p40 subunits, we confirmed that IL-23 heterodimer was indeed produced in acutely infected lungs, and that their levels as measured by ELISA were significantly diminished in the absence of eosinophils (Fig 2D). In conclusion, eosinophils are the predominant local source of IL-23 in this acute aspergillosis model.

Given that IL-23 can induce and augment IL-17 production, we next looked at whether the low IL-23 levels found in eosinophil-deficient ΔdblGATA-1 resulted in reduced IL-17 production by inflammatory cells recruited to the lungs following *A*. *fumigatus* challenge. However, expression of IL-17AF by macrophages, inflammatory monocytes, neutrophils and non-myeloid (CD11b^-^, CD11c^-^, Ly6G^-^) cells was not significantly different comparing lung cells from wild-type and ΔdblGATA-1 mice (S1 Fig).

### Localization of IL-23p19 and IL-17A in pulmonary eosinophils by confocal microscopy

**Fig 3:**
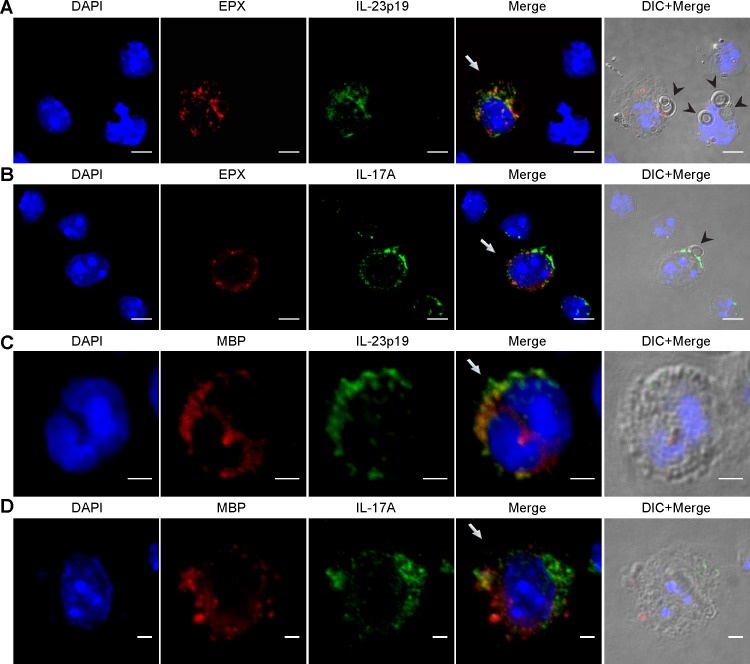
Confocal microscopy demonstrates intracellular IL-23p19 and IL-17A in eosinophils. Mice were challenged with *A*. *fumigatus*, treated with monensin, subjected to bronchoalveolar lavage and the lavaged cells analyzed by confocal microscopy as in *Methods*. Eosinophils were identified by staining for EPX (A and B) or MBP (C and D). Cells were analyzed 8 (A and C) and 54 (B and D) hours post-infection. The Merge images superimpose all 3 fluorescent images. The Merge+DIC images are the Merge images superimposed on differential interference contrast (DIC) images. Arrows and arrowheads point to eosinophils and conidia, respectively. Scale bars, 5 μm (A and B) and 2 μm (C and D). Photomicrographs are representative images from 3 independent experiments.

To confirm that eosinophils make IL-23p19 and IL-17A and to assess the intracellular location of these cytokines, we performed confocal microscopy on cells obtained by bronchoalveolar lavage 8 and 54 hours post-infection with *A*. *fumigatus* (Fig 3). Cells were identified as eosinophils if they had granules that stained positive for eosinophil peroxidase (EPX, Fig 3A and 3B) or major basic protein (MBP, Fig 3C and 3D). Staining for IL-23p19 and IL-17A was observed in 97.7 ± 1.2% (n = 130 eosinophils counted on 7 cytospin slides) and 90.9 ± 2.6% (n = 66 eosinophils counted on 5 cytospin slides), respectively, of the eosinophils examined. Moreover, IL-23p19 staining was observed exclusively in eosinophils (Fig 3A). Zero out of 711 cells that did not stain for EPX or MBP examined by confocal microscopy exhibited positive staining for IL-23p19. In contrast, IL-17A staining was more promiscuous (Fig 3B). Neither cytokine co-localized on a consistent basis with MBP- or EPX-positive granules.

### Eosinophils associate with and kill *A*. *fumigatus*

**Fig 4:**
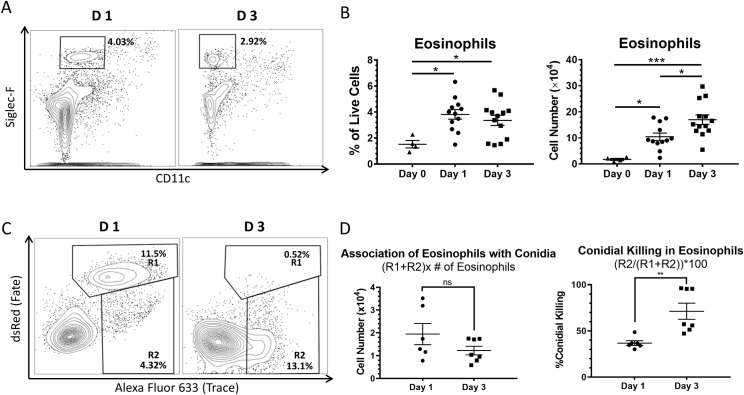
Eosinophils associate with and kill *A*. *fumigatus* conidia. BALB/c mice were infected with 5x10^7^ FLARE conidia. One and three days post-infection, lung single cell suspensions were assessed for eosinophils (Siglec-F^+^ CD11c^-^), and their capacity to associate with and kill conidia. (A) Representative FACS plots at days 1 and 3. Representative plots from 3 experiments with 12 mice in each group. (B) Left panel. Percentage of eosinophils among total live lung cells at day 0 (uninfected, ▲), day 1 (●) and day 3 (■) post-infection. Differences were statistically not significant (ns). Right panel. Total number of eosinophils in the lungs at day 0 (uninfected, ▲), day 1 (●) and day 3 (■) post-infection. Plot from 3 separate experiments with a total of 12 mice per group. (C) Eosinophils gated as in A were analyzed for whether they had associated with conidia and if so whether the conidia were alive (gate R1) or dead (gate R2). Representative FACS plots at days 1 and 3 are shown. Plot representative of 3 separate experiments with 6 mice per group. (D) Left panel. The number of eosinophils associated with conidia was calculated by multiplying the sum of the percentages in gates R1 and R2 by the total number of eosinophils. Significant differences between days 1 and days 3 were not seen. Right panel. Conidial killing by eosinophils was assessed at days 1 and 3 using the FLARE assay. Percent conidial killing was calculated by the quotient of events in gate R2 and the sum of R1 and R2 multiplied by 100. An increase in conidial killing was observed from day one (mean = 36.9%) to day three (mean = 68.6%) post-infection. Data were statistically analyzed using t-tests, * = p < 0.05, ** = p < 0.01, ns = p > 0.05. Plot from three separate experiments with a total of 6 mice per group.

To further characterize the innate eosinophil response to acute pulmonary aspergillosis, we assessed their recruitment over the first three days of infection. From day 1 to day 3 post-infection, the percentage of total leukocytes in the lungs that were eosinophils (Siglec-F^+^ CD11c^-^) did not significantly change (Fig 4A). However, an increase in the number of eosinophils was observed (Fig 4B). Consistent with data from Mesnil et al. [25], uninfected BALB/c mice had detectable but relatively low numbers of resident eosinophils (Fig 4B).

To quantitatively assess the ability of eosinophils to associate with and kill *A*. *fumigatus* conidia *in vivo*, we used the Fluorescent Aspergillus Reporter (FLARE) assay developed by Jhingran et al. [26]. As this assay does not distinguish between conidia that are internalized from those that are surface-bound, the term “association” is used here to describe binding with or without phagocytosis. The FLARE assay exploits the instability of fluorescent proteins (i.e., dsRed) in denaturing environments such as phagolysosomes to assess the fate (i.e., viability) of conidia associated with a leukocyte of interest. Labeling dsRed-expressing conidia with a trace fluorophore, here we used Alexa Fluor 633, allows for the detection of live conidia (dsRed^+^ AF 633^+^) and dead conidia (dsRed^-^ AF633^+^).

Using the FLARE assay, the capacity of eosinophils to associate with and kill conidia in the lungs of infected BALB/c mice was assessed on days one and three post-infection. Association was calculated by adding the proportion of cells associated with live conidia (Gate R1) to the proportion of cells associated with dead conidia (Gate R2). The number of eosinophils found to be associated with conidia remained constant from day one to day three post-infection (Fig 4C and 4D). However, a significant increase in conidial killing was observed from day one (36.9%) to day three (68.6%) post-infection (Fig 4C and 4D).

### Eosinophils enhance inflammatory monocyte recruitment and lung macrophage population expansion

**Fig 5:**
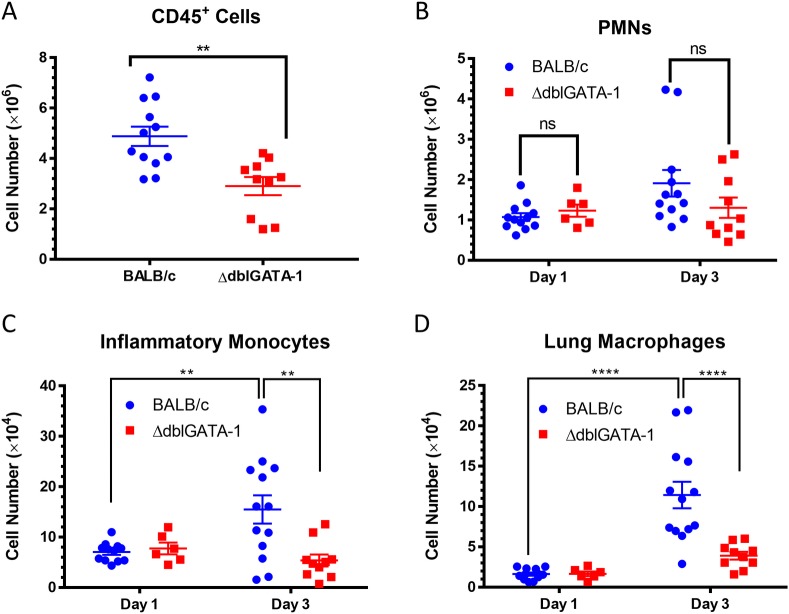
Eosinopenic mice show decreased recruitment of inflammatory monocytes to the lungs and reduced expansion of lung macrophages following challenge with *A*. *fumigatus*. Lung single-cell suspensions from BALB/c (blue circle) and ΔdblGATA-1 (eosinopenic; red square) mice infected with 5x10^7^ AF293 conidia were made one and three days post-infection and assessed for leukocyte populations. (A) Lack of eosinophils correlates with lower levels of CD45^+^ cells in the lungs three days after infection with 5x10^7^ conidia. ** = p < 0.001. (B) Neutrophil numbers in the lungs do not significantly differ comparing day 1 to day 3 post-infection. Furthermore, lack of eosinophils does not significantly affect neutrophil recruitment. p>0.05 (ns). (C,D) Inflammatory monocyte and lung macrophage numbers failed to increase in the absence of eosinophils. ** = p < 0.01, **** = p < 0.0001 Plots are aggregates from 3 separate experiments with 6–13 mice per group.

We next considered whether eosinophils also could contribute to host defenses against aspergillosis by directly or indirectly recruiting other phagocytes to the site of infection. Therefore, we assessed the number of leukocytes (CD45^+^ cells) three days after infection with 5x10^7^ *A*. *fumigatus* conidia in the lungs of BALB/c and ΔdblGATA-1 mice. We found that BALB/c mice had approximately 2x10^6^ more CD45^+^ cells than ΔdblGATA-1 mice (Fig 5A). This difference could not be accounted for by the lack of eosinophils in ΔdblGATA-1 mice, as their numbers averaged 1.7x10^5^ ± 0.2x10^5^ in BALB/c mice at the same 3 day time point (Fig 4B).

Considering that eosinophils were found to be sources of IL-23 and IL-17 in acute aspergillosis (Figs 1 and 2) and as these cytokines play a significant role in the recruitment of neutrophils [27], we evaluated the degree of lung neutrophilia one and three days post-infection in BALB/c and ΔdblGATA-1 mice. We found no differences in neutrophil numbers between groups (Fig 5B**)**. However, when we assessed the number of inflammatory monocytes (Siglec-F^-^ CD11b^+^ CCR-2^+^) and lung macrophages (Siglec-F^+^ CD11c^+^), we found that both phagocyte populations increased from day one to day three in BALB/c mice but not in ΔdblGATA-1 mice (Fig 5C and 5D). In fact, at three days post-infection, ΔdblGATA-1 mice had fewer inflammatory monocytes and macrophages than BALB/c mice. These data suggest that lack of eosinophils disrupts inflammatory monocyte recruitment, which then hampers lung macrophage expansion as inflammatory monocytes differentiate into macrophages and DCs at sites of inflammation [28,29]. Nevertheless, percent killing of conidia by inflammatory monocytes and neutrophils was not significantly different when comparing BALB/c with ΔdblGATA-1 mice (S2 Fig).

### Eosinopenia renders mice more susceptible to acute aspergillosis

**Fig 6:**
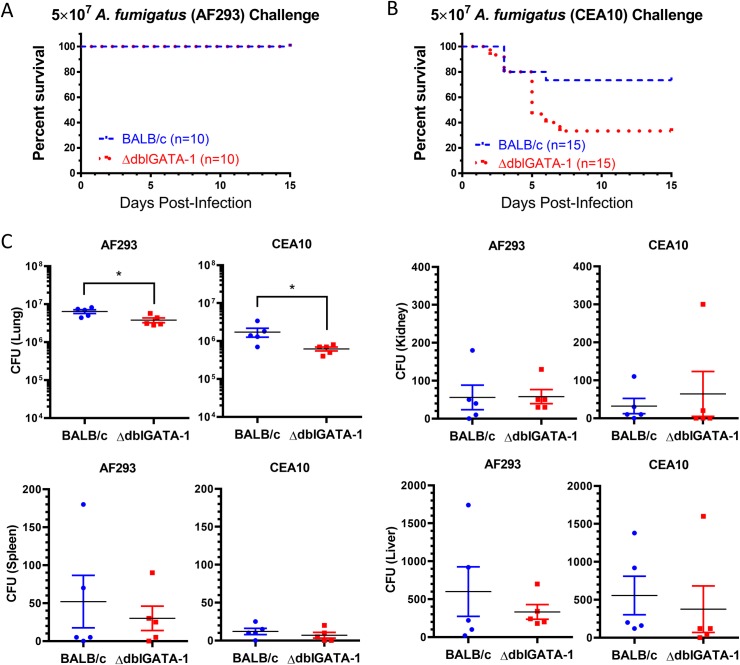
Eosinophils protect against mortality in acute infection with the CEA10 strain but not with the AF293 strain of *A*. *fumigatus*. Mortality study of BALB/c (blue) and ΔdblGATA-1 (red) mice infected with 5x10^7^ conidia (A) AF293 conidia and (B) CEA10 conidia. Kaplan-Meyer curves show combined data from two (for AF293) and three (for CEA10) separate experiments, each with 5 mice per group. * = p < 0.05. (C) Organ CFU 2d post infection of BALB/c and ΔdblGATA-1 mice with 5x10^7^ conidia of the indicated *A*. *fumigatus* strain. Data are from 2 separate experiments, one with 3 mice per group and the other with 2 mice per group. * = p < 0.05.

Infecting BALB/c and ΔdblGATA-1 mice with 5x10^7^ *A*. *fumigatus* conidia from the AF293 strain rendered no casualties (Fig 6A). However, after infection with the same inoculum of the CEA10 strain, which has been shown to be a more virulent strain in mouse models of acute aspergillosis [30,31,32,33,34], ΔdblGATA-1 mice had significantly higher mortality compared with BALB/c mice (Fig 6B).

To examine why lack of eosinophils was associated with increased mortality, we looked at CFUs in lungs, kidneys, liver and spleen 2d post infection. ΔdblGATA-1 mice had a modest but statistically significant decrease in lung CFUs following challenge with both *A*. *fumigatus* strains (Fig 6C). Extrapulmonary dissemination was observed but did not significantly differ when comparing BALB/c and ΔdblGATA-1 mice (Fig 6C). The paradoxical decreased number of CFUs in the lung in the ΔdblGATA-1 mice suggested that the mice could be succumbing to a robust inflammatory response rather than uncontrolled infection. To study this further, we looked at lung histopathology in BALB/c and ΔdblGATA-1 mice 2d following infection with the AF293 and CEA10 strains of *A*. *fumigatus* (S3 Fig**)**. In both groups, H&E stained sections revealed inflammatory cells in the airways and mainly nearby airspaces. The inflammatory cells were predominantly composed of histiocyctes admixed with sparse lymphocytes and neutrophils. Eosinophils were also noted in the lung sections from the BALB/c but not ΔdblGATA-1 mice. Gomori's methenamine silver (GMS) stained sections demonstrated numerous conidia and hyphae in the inflamed areas of the lungs. Interestingly, the lungs from CEA10 infected wild-type and ΔdblGATA type mice revealed relatively more hyphal growth compared to those infected with AF293. However, despite the great difference in fungal germination, histology at two days post-infection did not demonstrate discernable differences in the inflammatory response when comparing the wild-type and eosinopenic mice infected by the two *A*. *fumigatus* strains (S3 and S4 Figs).

### Eosinophils co-produce IL-23p19 and IL-17AF in allergic asthma

**Fig 7:**
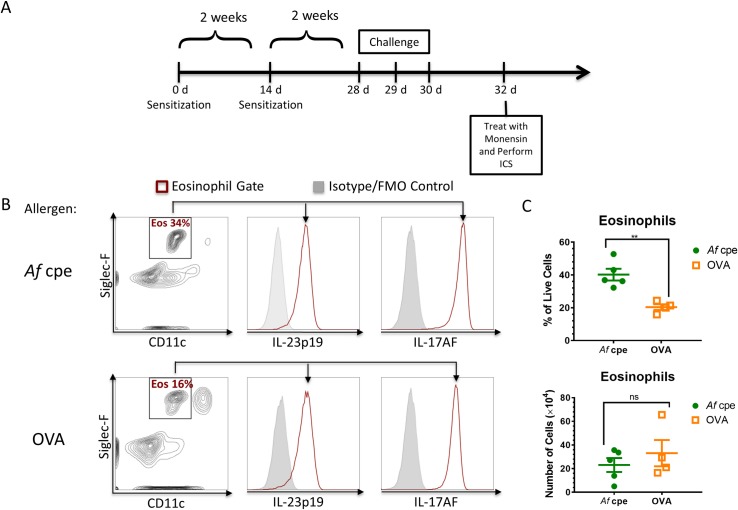
Eosinophils co-produce IL-23p19 and IL-17AF in two different models of asthma. (A) Schematic of sensitization and challenge timeline for asthma models. C57Bl/6 mice were sensitized with either 20 ug of OVA in alum or 200 ug of *A*. *fumigatus* crude protein extracts (*Af* cpe) by IP injections twice in two week intervals. Two weeks after the second sensitization, mice were challenged with either OVA or *Af* cpe by aerosol exposure on days 28, 29, and 30 after the first sensitization. Two days after the last challenge, mice were treated with 500 ug of monensin by IP injection. Six hours after the monensin treatment, ICS was performed in lung single cell suspensions. (B) Lung cells in the eosinophil (Siglec-F^+^ CD11c^-^) gate were examined for production of IL-23p19 and IL-17AF. Histograms show the shift in signal intensity for each cytokine compared to FMO controls. (C) Percent and number of eosinophils in the lungs of *Af* cpe and OVA sensitized mice. Although eosinophils made up a significantly higher proportion of lung leukocytes in mice sensitized and challenged with *Af* cpe compared to the OVA group, the number of eosinophils did not differ statistically between the two groups (n = 5). ** = p < 0.01; ns = p > 0.05.

As eosinophils are thought to drive many of the pathological findings in allergic asthma, and as increased levels of IL-17 have recently been correlated with severity of asthma symptoms [35], we next assessed whether eosinophils can also produce IL-23p19 and/or IL-17 in murine models of allergic asthma. Two established models of acute allergic asthma were chosen. In the first, mice were sensitized and challenged with *A*. *fumigatus* crude protein extracts (*Af* cpe) [36]. In the second model, sensitization was achieved with OVA admixed with aluminum hydroxide and challenge was performed with aerosolized OVA [37] (Fig 7A). In each of the allergic asthma models, eosinophils produced both IL-23p19 and IL-17AF (Fig 7B). In *Af* cpe sensitized and challenged mice, a significantly higher proportion of lung leukocytes were identified as eosinophils when compared to the OVA asthma model. Despite this difference, in both models a similar absolute number of eosinophils were recruited to the lungs (Fig 7C).

**Fig 8:**
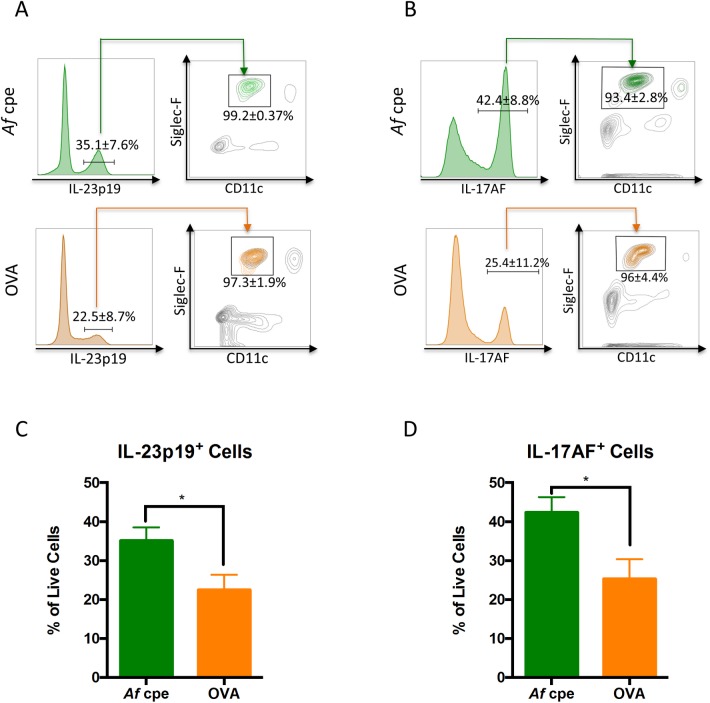
Eosinophils are a significant source of local IL-23 and IL-17AF in asthma. C57Bl/6 mice were sensitized and challenged with *Af* cpe or OVA as in Fig 7A. Lung cells from monensin-treated mice were then analyzed by ICS. (A and B) IL-23p19^+^ cells (A) and IL-17AF^+^ cells were analyzed for expression of Siglec-F and CD11c. Boxes denote the eosinophil (Siglec-F^+^ CD11c^-^) gate. The numbers under the box are mean ± SD (n = 5 mice per group). The vast majority of cells that stained positive for IL-23p19 and IL-17AF were in the eosinophil gate. (C,D) Percent of live lung cells that stained for IL-23p19 (C) and IL-17AF (D) after *Af* cpe and OVA sensitizations. Sensitization with *Af* cpe induced higher levels of IL-23p19^+^ and IL-17AF^+^ cells than OVA sensitization. * = p < 0.05.

Greater than 90% of IL-23p19^+^ and IL-17AF^+^ cells were identified as eosinophils regardless of sensitizing allergen (Fig 8A and 8B). On average, 35.1% of lung cells produced IL-23p19 in the *A*. *fumigatus* asthma model, compared to 22.5% in the OVA model (Fig 8A and 8C). Sensitization with *A*. *fumigatus* cpe elicited also elicited a higher percentage of IL-17AF^+^ cells compared with OVA sensitization (Fig 8B and 8D). Lung macrophages, inflammatory monocytes and neutrophils also produced IL-17AF, although at levels below those seen in eosinophils (S5 Fig). However, among the myeloid populations studied, only eosinophils were significant producers of IL-23p19 (S6 Fig).

## Discussion

The data presented here establish a new paradigm in acute and allergic aspergillosis whereby eosinophils act not only as effector cells but also as immunomodulatory cells driving the IL-23/IL-17 axis and contributing to inflammatory cell recruitment. Thus, following acute challenge with *A*. *fumigatus* conidia, eosinophils associated with and killed *A*. *fumigatus* conidia and ΔdblGATA-1 mice deficient in eosinophils were hypersusceptible to invasive aspergillosis. In acute and allergic models of aspergillosis, pulmonary eosinophils were found to be prominent sources of IL-23p19 and IL-17. Finally, following acute *A*. *fumigatus* challenge, ΔdblGATA-1 mice had fewer total leukocytes, inflammatory monocytes and lung macrophages compared to wild-type mice.

Although over 95% of the eosinophils were found to be positive for IL-23p19 and IL-17A (Figs 1 and 2), only 18% and 8% of eosinophils were found to associate with conidia one and three days post-infection, respectively (Fig 4). This disparity suggests that direct association with *A*. *fumigatus* is not necessary for the production of IL-23p19 or IL-17A. The milieu in which the eosinophils are stimulated appears to be important as co-incubation of bone-marrow derived eosinophils with *A*. *fumigatus* conidia or zymosan failed to elicit IL-23 or IL-17 production as measured by ELISA and ICS. These findings underscore the plasticity of eosinophils; as with other immune cells, subsets appear to exist. For example, in the small intestine, eosinophils constitutively secrete IL-1 receptor antagonist which downregulates T_H_17 cells [38] whereas in response to parasite challenge eosinophils promote T_H_2 immunity [39]. Future work should focus on determining the extrinsic and intrinsic molecular pathways that stimulate eosinophils to produce IL-23 and IL-17.

IL-23p19 and IL-17 production was observed in pulmonary eosinophils recruited to the lungs not only in models of acute and allergic aspergillosis but also in a model of allergic asthma induced by sensitization to OVA (Figs 7 and 8). As eosinophils have been reported to express IL-23R [40], this suggests an autocrine amplification loop may be occurring whereby eosinophils release IL-23 which directly feeds back on itself via IL-23R to enhance IL-17 production. As a precedent, eosinophils express IL-5R and have IL-5 in their granules [40]. Other IL-23-producing cell types, including alveolar macrophages, have been found to produce IL-17 under defined conditions [20,41]. We postulate that eosinophils may be contributing to the IL-23/IL-17 axis under a broad spectrum of disease states. In support of this hypothesis, three other groups have reported evidence for eosinophil-derived IL-17 [42–43]. Using a reporter IL-17A-EGFP mouse line, Shimura et al. demonstrated that EGFP^+^ eosinophils in the peritoneum 6 hours following an IP LPS injection [43]. In humans, Molet et al. demonstrated IL-17 gene and protein expression in eosinophils obtained from the sputum, bronchoalveolar lavage fluid and blood of asthmatic subjects [42]. Lastly, Kobayashi et al. detected IL-17 in the supernatants of human eosinophils stimulated with monosodium urate crystals [44].

IL-23 is a key cytokine promoting and maintaining IL-17-producing cells, particularly T_H_17 cells [11,45]. Remarkably, whereas dendritic cells and macrophages are thought to be the primary sources of IL-23 in tissue [45,46]; in our aspergillosis models we found eosinophils were the major cell type in the lungs producing IL-23. Thus, in the acute aspergillosis model and both allergic models, the cells that stained the brightest for IL-23p19 were nearly all eosinophils. Their identity as eosinophils was confirmed by sorting and by the loss of this population in the ΔdblGATA-1 mice. Significantly, following challenge with *A*. *fumigatus* conidia, levels of the functional IL-23 heterodimer were dramatically reduced in the BALF of mice that lacked eosinophils compared to wild-type controls. Two IL-23p19 antagonists, tildrakizumab and guselkumab, are undergoing clinical trials for the treatment of autoimmune and allergic disease [46]. For the subset of these diseases in which eosinophilia is seen, our data suggest that targeting eosinophils could have therapeutic efficacy while avoiding potential toxicities such as impaired host defenses to infections that could occur following global inhibition of the IL-23/IL-17 axis [46].

In mouse models of asthma, the absence of eosinophils correlates with decreased airway hyperresponsiveness (AHR) and decreased mucus production [47]; these findings are also found in mice lacking IL-17 signaling [48,49]. Another example of functional overlap is the finding that IL-17 signaling and eosinophil co-culture with epithelial cells have each been shown to up-regulate Mucin 5AC, a mucin protein produced by bronchial airway goblet cells [48,50,51]. The convergence between IL-17 and eosinophils found in our models of allergic asthma point to a novel mechanism by which eosinophils contribute to the pathogenesis of asthma in a manner that is independent of T_H_2-driven inflammation. If this phenomenon holds up in humans, as is supported by the data of Molet et al. [42], it could be of use to better stratifying asthma type, as heterogeneity in this disease has prevented the success of some targeted asthma therapies [48,52].

Clinically, asthma features episodic shortness of breath accompanied by wheezing due to AHR. However, it has been increasingly recognized that a heterogeneous group of immunological processes drives these hallmark symptoms [48,53]. Individualizing therapy based on the immunological drivers has the potential to improve therapeutic outcome, which is relevant considering many patients fail to respond to standard treatment [53,54]. Stratification based on the predominant cellular infiltrate found in induced sputum has delineated four distinct types of asthma: eosinophilic, neutrophilic, mixed granulocytic and paucigranulocytic [55]. It will be interesting to assess the presence of IL-23^+^ IL-17^+^ eosinophils in each of these types, as even in neutrophilic and paucigranulocytic asthma a small number of eosinophils is present [55].

While eosinophils act as drivers of inflammation and tissue remodeling in asthma [39,52,56], they play a protective role in models of acute pulmonary aspergillosis. Eosinopenic mice challenged with a relatively virulent strain of *A*. *fumigatus* had increased mortality compared to wild-type mice (Fig 6) despite similar CFUs and no striking differences in lung pathology (Figs 6, S3 and S4). In contrast, Lilly et al. found *Aspergillus*-infected mice lacking eosinophils had higher fungal burdens, as measured by *A*. *fumigatus* 18S rRNA and fungal germination [10].

How eosinophils protect hosts still needs to be more fully defined and may be multifactorial. Eosinophils directly kill *A*. *fumigatus* in vivo (Fig 4) and by contact-dependent mechanisms in vitro [10]. Moreover, lysates containing eosinophil granule proteins inhibit fungal germination [10]. However, we also found eosinophils act indirectly by promoting the recruitment of inflammatory monocytes and macrophages to the lungs after infection (Fig 5). Alveolar macrophages and inflammatory monocytes kill *A*. *fumigatus* conidia [29,57]. In addition, mice depleted of inflammatory monocytes are vulnerable to *A*. *fumigatus* infection [29]. Interestingly, ΔdblGATA-1 mice did not have a defect in neutrophil recruitment to the lung following *A*. *fumigatus* challenge (Fig 5 and [10]). Moreover, in a neutropenia mouse model of aspergillosis that features repeated aspiration of conidia from an unusual strain of *A*. *fumigatus* containing high cell wall chitin, decreases in fungal burden and mortality were observed in ΔdblGATA-1 mice [9].

In the context of the damage-response framework of microbial pathogenesis [58], we posit that eosinophils and eosinophilic production of IL-23 and IL-17 are beneficial in invasive aspergillosis but detrimental in allergic disease. Future studies will test this hypothesis and examine the spectrum of diseases under which eosinophils produce IL-23 and IL-17. These data could help inform clinical studies using monoclonal antibodies and other immunomodulators that target eosinophils and the IL-23/IL-17 axis.

## Materials and Methods

### Mice

Six- to eight-week old C57Bl/6, BALB/c, ΔdblGATA-1 mice in the BALB/c background, and IL-17A^GFP/GFP^ mice in the C57Bl/6 background were obtained from Jackson Laboratories and bred in pathogen-free conditions at the University of Massachusetts Medical School (UMMS).

### Ethics statement

The mouse studies were performed in accordance with protocol #1802–15 approved by the UMMS Institutional Animal Care and Use Committee. In addition, all mouse studies were carried out in accordance with the recommendations in the Guide for the Care and Use of Laboratory Animals of the National Institutes of Health.

### *Aspergillus fumigatus* culture and murine acute pulmonary aspergillosis model

*A*. *fumigatus* from the AF293 strain was obtained from the Fungal Genetics Stock Center (Manhattan, KS). AF293 was grown from frozen stocks on Sabouraud-dextrose agar (Remel) slants [59,60]. The CEA10 strain was a generous gift from Robert Cramer (Geisel School of Medicine, Hanover, NH) and grown as described [61]. Conidia were harvested by vortexing slants with PBS containing 0.01% Tween-20 (Thermo-Fisher) and filtering the suspension twice through a 30 μm nylon mesh folded over a conical tube. Suspensions were then washed three times with 0.01% Tween-PBS, and re-suspended in the same solution at a concentration of 6.67x10^8^ conidia/mL. Mice were infected via the oro-tracheal (OT) route with 75 μL of conidial suspension, so that each mouse received 5x10^7^ conidia. OT infection was facilitated by anesthetizing mice with isoflurane (Piramal Healthcare). Organ CFUs were enumerated as in our previous studies [62,63] except the resected organs were homogenized and diluted in PBS containing 0.01% Tween-20. The lower limit of detection was 10 CFU/organ.

### Generation of bone marrow-derived eosinophils (BM-Eos)

Bone marrow cells were isolated from murine femurs and differentiated as described previously [64]. Briefly, BM cells were subjected to a gradient with Histopaque 1083 (Sigma). Interphase cells were washed twice to remove any leftover Histopaque and cultured in Isocove’s modified Dulbecco’s medium (IMDM; Life Technologies) supplemented with 10% FBS (Tissue Culture Biologicals), 100 U/mL penicillin and 100 U/mL streptomycin, 1X Glutamax (Life Technologies) and 2 μL of β-mercaptoethanol (Life Technologies). The first four days of culture, cells were stimulated with 100 ng/mL of murine stem cell factor (mSCF) (PeproTech) and 100 ng/mL of murine FMS-like tyrosine kinase 3 ligand (mFLT3L) (PeproTech). The following ten days, cells were stimulated with 10 ng/mL IL-5 (R&D). Cells were fed every other day. Differentiation was confirmed by flow cytometry with rat mAb against murine Siglec-F-BV421 (BD Biosciences).

### BM-Eos stimulation

BM-Eos were stimulated with live or heat-killed *A*. *fumigatus* AF293 conidia, zymosan (10–100 μg/mL; Sigma), lipopolysaccharide (LPS, 100 ng/mL; Sigma) and several cytokines at different concentrations and combinations for lengths of time ranging from two hours to two days. Cytokines used to stimulate BM-Eos included IL-5 (10 ng/mL), IL-1β (1–10 ng/mL; R&D), GM-CSF (10 ng/mL; PeproTech), IL-23 (10 ng/mL; eBiosciences), IL-17E (0.1–10 ng/mL; R&D), IL-17AA (0.1–10 ng/mL; R&D), PGE_2_ (10^−5^–10^−3^ M; Cayman Chemicals), transforming growth factor-β (TGF-β) (1–10 ng/mL; eBiosciences), and IL-6 (10–100 ng/mL; eBiosciences). When co-incubated with live *A*. *fumigatus* conidia, cultures were supplemented with 0.5 μg/mL of voriconazole (Sigma) to prevent fungal overgrowth. Following stimulation, supernatants were collected for ELISA. In some experiments, cells were co-incubated with 1 μM monensin in the last 5 hours of stimulation, and intracellular cytokine staining was performed as described below.

### Fluorescent Aspergillus Reporter (FLARE) conidia

FLARE conidia were created as described [26,65]. Briefly, 5x10^8^ dsRed conidia (AF293) per mL were incubated with 0.5 mg/mL 6-((6-((Biotinoyl)Amino)Hexanoyl)amino)Hexanoic Acid, Sulfosuccinimidyl Ester, Sodium Salt (Biotin-XX, SSE) (ThermoFisher) in 50 mM carbonate buffer (Sigma), pH 8.3 at 4°C for 2 h in a tube rotator. Excess Biotin-XX, SSE was washed off with 0.1 M Tris-HCl pH 8.0 (Sigma); then conidia were incubated with 0.02 mg/mL streptavidin conjugated to Alexa Fluor 633 (Life Technologies) away from light at room temperature for 30 minutes. Labeling was confirmed by flow cytometry prior to infecting animals.

### Murine acute allergic asthma model

C57Bl/6 mice were sensitized two times by IP injection of 20 μg of OVA (Sigma-Aldrich) in 100 μL of Imject Alum (Thermo Scientific) or 200 μg of *A*. *fumigatus* crude protein extracts (*Af* cpe) (Greer Laboratories). Sensitization occurred two weeks apart. On days 28, 29 and 30 mice were challenged with aerosolized 1% OVA or 0.25% *Af* cpe in saline respectively for 20 minutes as described [36].

### Intracellular cytokine staining, sorting and flow cytometry

ICS was performed as previously described [66]. Briefly, mice were treated with 500 μg of monensin (Sigma-Aldrich) by IP injection, 2 or 48 h after infection with 5x10^7^ conidia, or 48 h after the last challenge in the allergic asthma models. Six hours after monensin treatment, lung single-cell suspensions were prepared using MACS lung dissociation kit as described by the manufacturer (Miltenyl Biotec). Single-cell suspensions were enriched for leukocytes using a Percoll (GE Healthcare) gradient [67]. Interphase cells were collected, counted with the aid of a hemocytometer, and then co-incubated with rat anti-mouse CD16/CD32 monoclonal antibody (mAb) 2.4G2 (BD Pharmingen) to block F_c_ receptors in accordance with manufacturer’s directions. Surface antigens were then stained with antibodies listed in Table 1 and with Fixable Viability Dye eFluor 780 (eBioscience) or Live/Dead Blue (Life Technologies) for 30 minutes at 4 C. After two successive wash steps, lung leukocytes were fixed in 2% paraformaldehyde (Electron Microscopy Sciences) PBS solution for at least 15 minutes at 4 C. Fixed cells were permeabilized using Perm/Wash Buffer (BD Pharmingen) according to manufacturer instructions and then stained with antibodies listed in **Table 1**.

**Table 1:** Antibodies used in flow cytometry.

Surface Markers			
mAb	Clone	Manufacturer	Isotype
Ly6G-PE-CF594	1A8	BD Biosciences	Rat IgG2a
CCR-2-FITC	475301	R&D Systems	Rat IgG2b
CD11c-BV 570	N418	BioLegend	Armenian Hamster IgG
Siglec-F-BV 421	E50-2440	BD Biosciences	Rat IgG2a
CD11b-BUV 395	M1/70	BD Biosciences	Rat IgG2b
CD45-PerCP-Cy5.5	30-F11	BD Biosciences	Rat IgG2b
Intracellular Markers			
mAb	Clone	Manufacturer	Isotype
IL-23p19-eFluor 660	Fc23cpg	eBiosciences	Rat IgG1
IL-17A-PE-Cy7	TC11-18H10.1	BioLegend	Rat IgG1
IL-17AF-eFluor 660	B8KN8R	eBiosciences	Rat IgG2a

For sorting IL-23p19^+^ IL-17A^+^ cells, fixed lung leukocytes were only stained for intracellular cytokines, and then sorted with BD FACSVantage DV-1 Cell Sorter (UMass Medical School Flow Cytometry Core). FC data were acquired with a BD LSR II cytometer and analyzed using FlowJo X software (Tree Star Inc.). Gating was established using FMO controls containing isotype control mAb conjugated with the fluorophore corresponding to the missing antibody as described [68].

### Confocal microscopy

Mice were infected with 5 x 10^7^ *A*. *fumigatus* strain 293 conidia via oral-tracheal route. After two or 48 h, mice received 500 μg of IP monensin, and 6 h later the mice were euthanized and bronchoalveolar lavage fluid was collected. Cells were washed with PBS, counted, fixed with 2% paraformaldehyde buffered in PBS and washed an additional 3 times. The lavage cells were then cytospun onto slides (Shandon Cytoslide), permeabilized for 10 minutes in 0.1% Triton-X 100 in PBS, washed three times with PBS and blocked with 10% donkey serum in PBS for 1 h at room temperature. Cells were sequentially immunostained in 5% donkey serum with goat polyclonal antibody (4°C, 3 hours), Alexa fluor 594-labeled donkey anti-goat fab2 (room temperature, 1 hour), rabbit polyclonal antibody (4 hours, 4°C), and Alexa fluor 647-labeled donkey anti-rabbit fab2 fragment (45 minutes, room temperature). The specific antibodies used, including the control antibodies, are listed in Table 2. Cell nuclei were stained with 4’6-diamino-2-phenylindole (DAPI, 1ug/ml; Sigma) and the slides were mounted with ProLong Gold Antifade Mountant (Life Technologies). Images were acquired with a 63x oil immersion objective at maximum intensity projections in sequential scan mode with a laser confocal microscope (Leica SP8).

**Table 2:** Antibodies used in confocal microscopy.

Antibody	Label	Manufacturer/Catalog #
goat polyclonal anti-MBP	None	Santa Cruz/109316
goat polyclonal anti-EPX	None	Santa Cruz/19148
goat polyclonal IgG^*^	None	Santa Cruz/3887
donkey anti-goat fab2	Alexa fluor 594	Abcam/150140
rabbit polyclonal anti-IL-23p19	None	Abcam/45420
rabbit polyclonal anti-IL-17A	None	Abcam/79065
rabbit polyclonal IgG^**^	None	Abcam/27478
donkey anti-rabbit fab2	Alexa fluor 647	Abcam/181347

* control for anti-MBP and anti-EPX.

** control for anti-IL-23p19 and anti-IL-17A.

### Cytological staining

After sorting, cells were adhered to poly-L-lysine-coated slides (Sigma-Aldrich) by cytospinning 250 μL of cell suspension at 800 rpm for three minutes (Shandon Cytospin 2). Cells were dried on the slide, and then dipped in hematoxylin solution (Thermo-Fisher) for 30 seconds. Slides were washed in water then dipped in eosin (Thermo-Fisher) for one minute. Increasing concentrations of ethanol (95%-100%) were used to dehydrate slides for a total of two minutes. Finally, slides were dipped three times in Clear-Rite (Thermo Scientific) for one minute each time. Slides were mounted using Permount (Fisher Scientific).

### Cytokine quantification in bronchoalveolar lavage fluid (BALF) after infection

BALF from BALB/c and ΔdblGATA-1 mice infected as described above was collected after euthanasia at 60 h post-infection. Briefly, an 18 or 20 gauge plastic catheter (Temuro) was inserted into the trachea and lungs were flushed three times with 1 mL of PBS supplemented with cOmplete protease inhibitor cocktail (Sigma-Aldrich). BALF samples were flash frozen in dry ice and subsequently stored in -80°C. IL-23 heterodimer (IL-23p19/IL-12p40) was measured using R&D Quantikine ELISA kit.

### Statistical analysis

Statistical tests were performed using GraphPad Prism 6. The Student’s t-test was used to compare the means of two groups with the Bonferroni correction applied for multiple comparisons. For data sets where the mean of more than two groups was compared, 2-way ANOVA was performed using Tukey’s multiple comparison correction. In comparing Kaplan-Meyer survival curves, the Mantel-Cox test was used.

## Supporting Information

S1 Fig Cell subsets responsible for pulmonary IL-17AF production in wild-type and ΔdblGATA-1 mice following acute challenge with *A*. *fumigatus*. (DOCX)

S2 Fig In vivo killing of *A*. *fumigatus* conidia by inflammatory monocytes and neutrophils in BALB/c and ΔdblGATA-1 mice. (DOCX)

S3 Fig Histopathology of infected lungs. (DOCX)

S4 Fig Inflammation of infected lungs. (DOCX)

S5 Fig Different myeloid cell types contribute to IL-17AF production in models of allergic asthma. (DOCX)

S6 Fig Eosinophils constitute the main myeloid cell in the lungs that produces IL-23p19 in models of allergic asthma. (DOCX)
