# Supplementary material for: Central Role of IL-23 and IL-17 Producing Eosinophils as Immunomodulatory Effector Cells in Acute Pulmonary Aspergillosis and Allergic Asthma
Source: PLoS Pathog. 2017 Jan 17;13(1):e1006175. doi: 10.1371/journal.ppat.1006175 (PMC5271415; doi:10.1371/journal.ppat.1006175)
Supplement: S1 Fig — (DOCX) [file ppat.1006175.s001.docx]

**
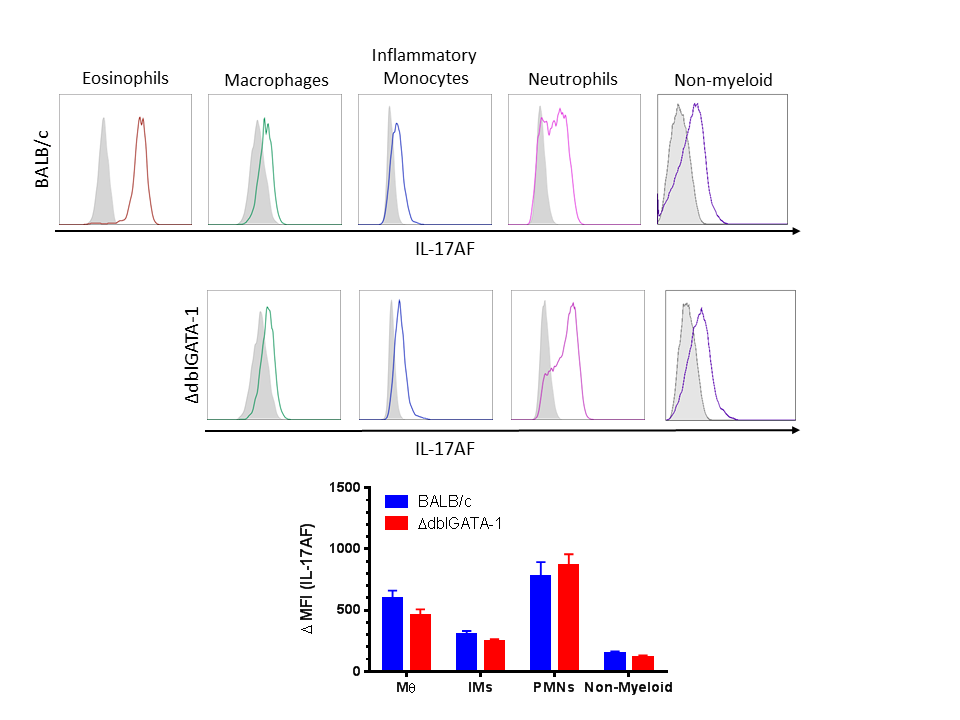
**

**Figure S1. Cell subsets responsible for pulmonary IL-17AF production in wild-type and ΔdblGATA-1 mice following acute challenge with *A. fumigatus*.** The methodology for infecting the mice, making single-cell suspensions, ICS and flow cytometry was the same as in Figures 1 and 2. In addition to their forward and side scatter characteristics, markers used to define cell types were as follows:

| ***Marker*** | ***Eosinophils*** | ***Neutrophils*** | ***Inflammatory Monocytes*** | ***Lung Macrophages*** |
| --- | --- | --- | --- | --- |
| Ly6G |  | ++ |  |  |
| CCR-2 | - | - | + |  |
| CD11c | - | - |  | + |
| Siglec-F | + | - | - | + |
| CD11b | + | + | + |  |

Non-myeloid cells were defined as cells which were negative for CD11b, CD11c and Ly6G. The top and middle panels show representative histograms. The shaded gray areas depict the isotype controls. The bottom panel shows the means ± SEM increases (compared with isotype controls) in IL-17AF median fluorescent intensity (ΔMFI) from 2 independent experiments each of which had 4-5 mice. There were no significant differences comparing wild-type and ΔdblGATA-1 mice for macrophages (MΦ), inflammatory monocytes (IM), neutrophils (PMNs) or non-myeloid cells.
