# Supplementary material for: Central Role of IL-23 and IL-17 Producing Eosinophils as Immunomodulatory Effector Cells in Acute Pulmonary Aspergillosis and Allergic Asthma
Source: PLoS Pathog. 2017 Jan 17;13(1):e1006175. doi: 10.1371/journal.ppat.1006175 (PMC5271415; doi:10.1371/journal.ppat.1006175)
Supplement: S2 Fig — (DOCX) [file ppat.1006175.s002.docx]

**
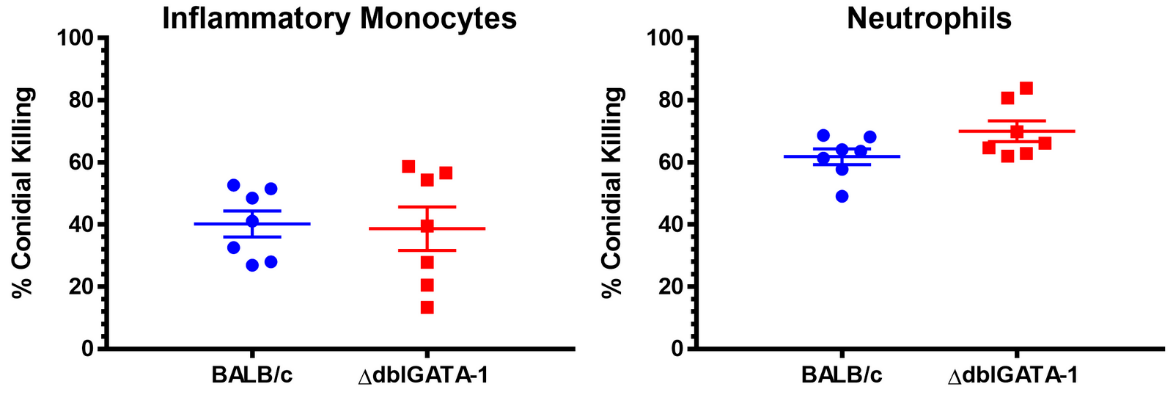
**

**Figure S2. In vivo killing of *A. fumigatus* conidia by inflammatory monocytes and neutrophils in BALB/c and ΔdblGATA-1 mice.** BALB/c and ΔdblGATA-1 mice were infected with 5x10^7^ AF293 FLARE conidia. Three days post-infection, lung single cell suspensions were made and the capacity of inflammatory monocytes and neutrophils to kill the conidia was assessed using the FLARE assay as in Figure 4. Inflammatory monocytes and neutrophils were identified by flow cytometry as in Figure 5. Data are from two independent experiments, one with 3 mice per group and the other with 4 mice per group. There were no significant differences comparing wild-type and ΔdblGATA-1 for either phagocyte population studied.
