# Supplementary material for: Central Role of IL-23 and IL-17 Producing Eosinophils as Immunomodulatory Effector Cells in Acute Pulmonary Aspergillosis and Allergic Asthma
Source: PLoS Pathog. 2017 Jan 17;13(1):e1006175. doi: 10.1371/journal.ppat.1006175 (PMC5271415; doi:10.1371/journal.ppat.1006175)
Supplement: S3 Fig — (DOCX) [file ppat.1006175.s003.docx]

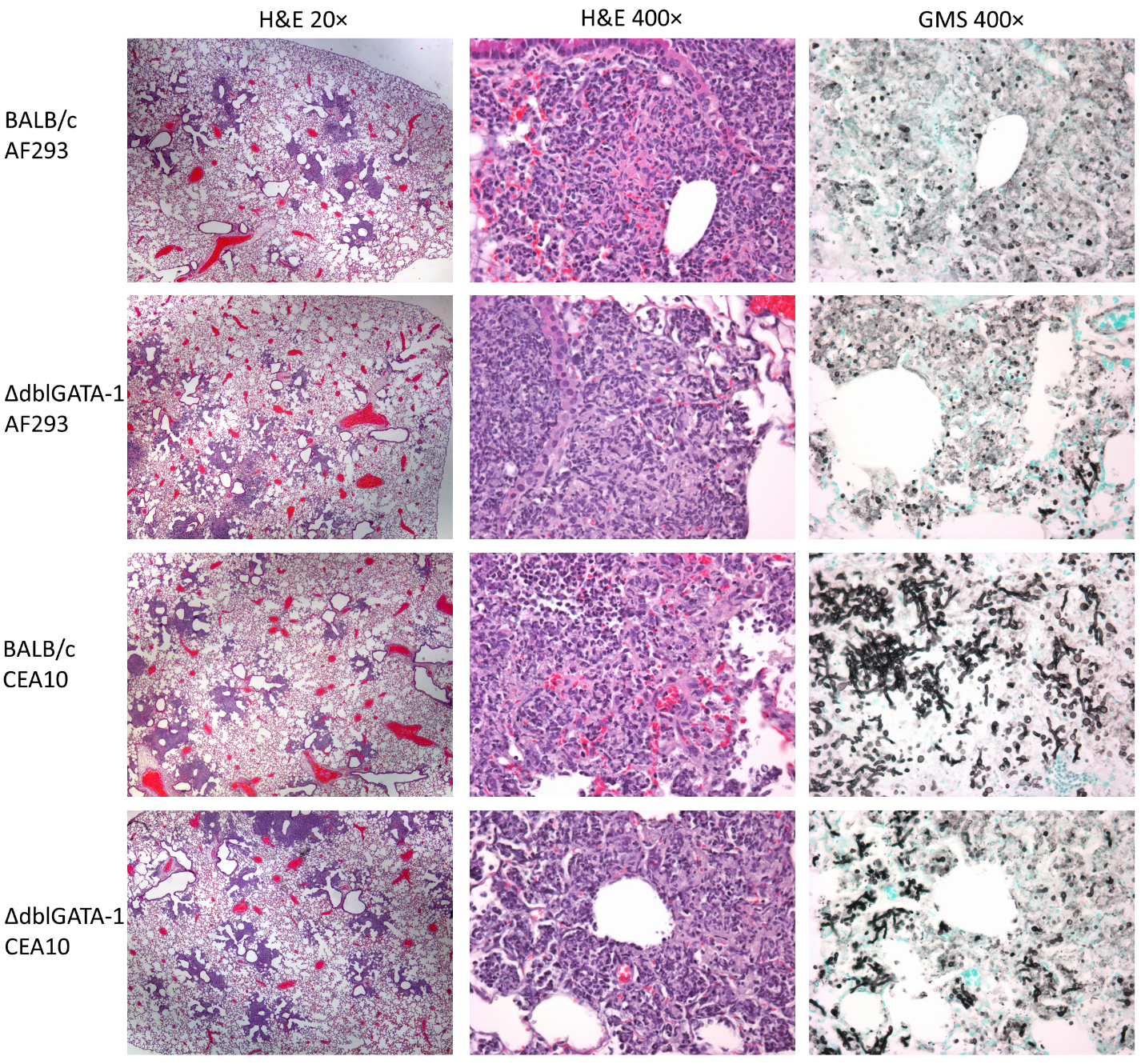


**Figure S3. Histopathology of infected lungs.** Wild-type BALB/c and ΔdblGATA-1 mice were infected via the orotracheal route with 5 x 10^7^ conidia of *A. fumigatus* strain 293 or CEA10. Two days post-infection, histopathology on infected lung tissue was performed following euthanasia with CO_2_, exposure of the trachea and inflation of the lungs with 4% buffered formalin. Lungs were then harvested, embedded in paraffin and 5 μm thick sections that included each of the lobes were cut. The slides were stained with hematoxylin and eosin (H&E) and Grocott’s methenamine silver (GMS, American MasterTech). H&E and GMS fields taken from areas of inflammation are shown. A total of five mice per group were studied in two independent experiments.
