# Supplementary material for: Central Role of IL-23 and IL-17 Producing Eosinophils as Immunomodulatory Effector Cells in Acute Pulmonary Aspergillosis and Allergic Asthma
Source: PLoS Pathog. 2017 Jan 17;13(1):e1006175. doi: 10.1371/journal.ppat.1006175 (PMC5271415; doi:10.1371/journal.ppat.1006175)
Supplement: S4 Fig — (DOCX) [file ppat.1006175.s004.docx]

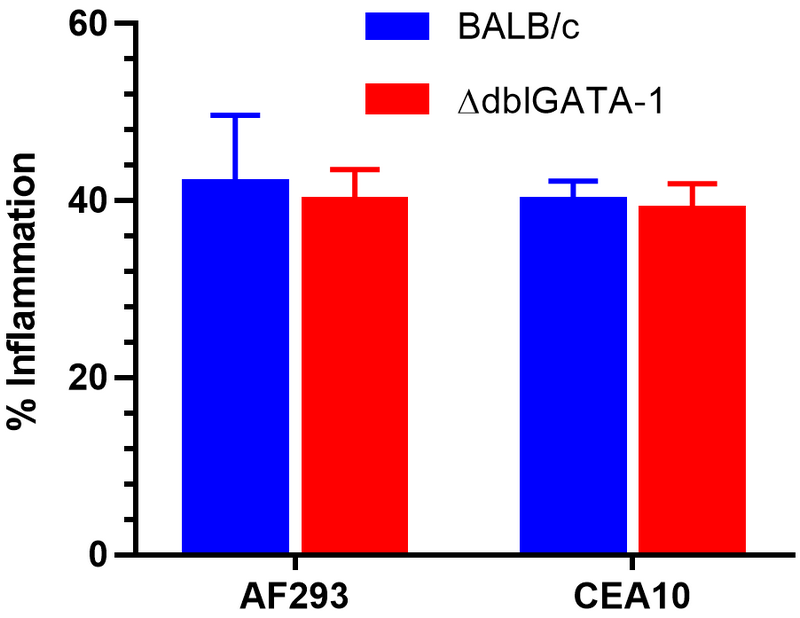


**Figure S4. Inflammation of infected lungs.** Pulmonary pathology was performed on wild-type BALB/c and ΔdblGATA-1 mouse lungs 2 days post-infection with 5 x 10^7^ conidia of *A. fumigatus* strain 293 or CEA10 as in Figure S2. The mean percentage of lung area involved by inflammation was estimated (in 5% increments) after looking at 5 H&E-stained sections of lung per mouse (one section from each lobe) at 20x final magnification. No significant differences in percent lung inflammation were seen when comparing any two groups. Data are means ± SEM of 5 mice per group.
