# Supplementary material for: Central Role of IL-23 and IL-17 Producing Eosinophils as Immunomodulatory Effector Cells in Acute Pulmonary Aspergillosis and Allergic Asthma
Source: PLoS Pathog. 2017 Jan 17;13(1):e1006175. doi: 10.1371/journal.ppat.1006175 (PMC5271415; doi:10.1371/journal.ppat.1006175)
Supplement: S6 Fig — (DOCX) [file ppat.1006175.s006.docx]

**
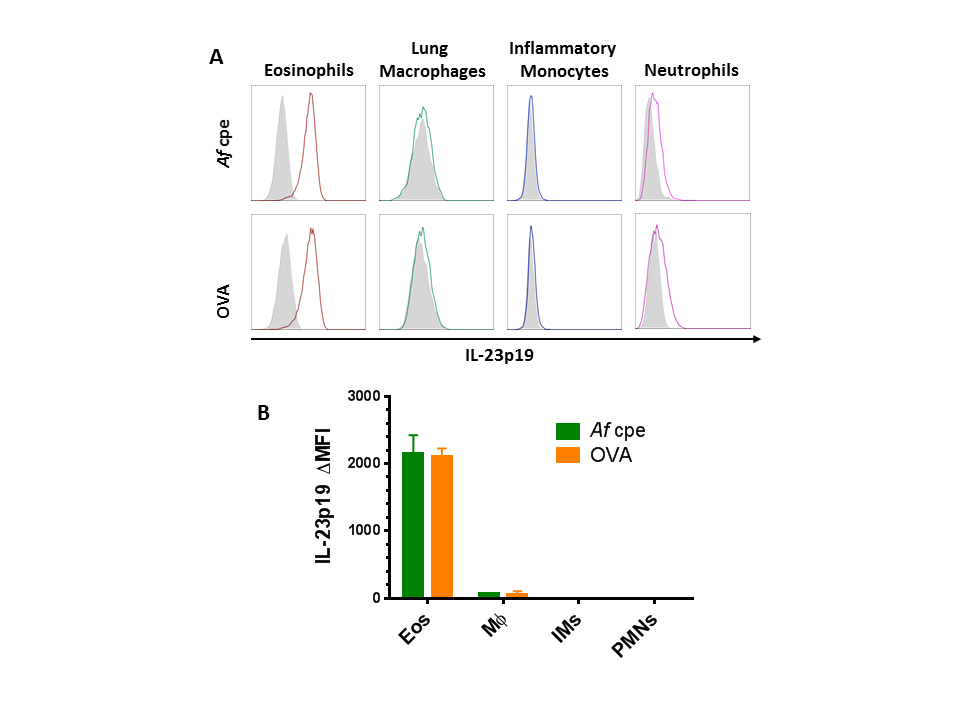
**

**Figure S6. Eosinophils constitute the main myeloid cell in the lungs that produces IL-23p19 in models of allergic asthma.** The methodology for the allergic sensitization model is shown in Figure 7A. The methodology for making single-cell suspensions, ICS and flow cytometry was the same as in Figures 1 and 2 and Supplemental Figure 1. In both *A. fumigatus* antigen (*Af* cpe) and OVA models of allergic sensitization, IL-23p19 is produced in significant amounts by eosinophils (eos) but not lung macrophages (MΦ), inflammatory monocytes (IMs) and neutrophils (PMNs).  **(A)** Representative histograms. The shaded gray areas depict the isotype controls. **(B)** Means ± SEM increases (compared to isotype controls) in IL-23p19 median fluorescent intensity (ΔMFI) for each myeloid cell population. p<0.001 comparing IL-23p19 ΔMFI for eosinophils with any other cell type following sensitization with either *Af* cpe or OVA. Data were analyzed by two-way ANOVA, employing Tukey’s multiple comparison test, n=5 mice per group.
